# Commentary: Problems With Police Reports as Data Sources: A Researchers' Perspective

**DOI:** 10.3389/fpsyg.2022.873235

**Published:** 2022-06-27

**Authors:** Stefan Schade, Markus M. Thielgen

**Affiliations:** Area of Study VIII—Social Sciences, Department I—University Education, Rhineland-Palatinate Police University, Büchenbeuren, Germany

**Keywords:** police reports, police officers' mindset, confirmation bias, memory malleability, research subject

## Introduction

Güss et al. (2020) recently published an article on problems with police reports as data sources from a researchers' perspective. Based on their research project on police reports, they reported their experiences with conducting research using this type of data, presenting problems they encountered. According to Güss et al., the first problem concerns the limited access to police reports, while the second problem arises from the poor quality of police reports. Their experiences are based in the United States, and it seems that using police reports as a data source did not meet the researchers' expectations. At first glance, one may assume that researchers would find a comparable situation regarding police reports in Germany (and probably in all locations).

## Police Data Access and Data Quality

The first problem highlighted by Güss et al. (2020) derives, not surprisingly, from access to police data in general. Most recently, Geugjes and Terizakis (2022) reported comparable experiences to Güss et al. (2020) from their own research project conducted in several federal states of Germany. Similarly to the United States, Germany is federally organized. By constitution (Art. 30 GG), the 16 federal states execute governmental authority by 16 state police forces and have different police laws and specific regulations that determine practical police work and consequently access to police data. Moreover, the willingness of police organizations to support external (independent) research seems to differ both between federal states and within federal states between local police departments. For instance, whether access to police data is permitted may depend on the openness of individual police leaders and the habitus of local police departments (Geugjes and Terizakis, 2022; Schöne and Herrnkind, 2022).

Research questions concerning the police have high public relevance. However, due to structural circumstances within the federal states of Germany, there is variation in data access and openness to research projects by the police leadership. For instance, the Rhineland-Palatinate police university may be considered as a more “integrated” part of the state police by police leaders themselves, rather than a separate administration. Here, it may be easier to obtain data access, if the police leadership and policymakers are convinced of the practical benefit of a research project. Problematically, data access and openness to research projects may depend on the organizational *non-scientific* leadership of the police or the interior ministries of the federal states. Even when research projects are welcomed, they may not be prioritized due to lack of personal and financial resources. Additionally, as Staller and Koerner (2022) noted, research and science may be used as value labels by the police leadership to highlight their own interests, rather than to solve relevant problems within the police. Thus, collaboration between independent researchers and police organizations may be a promising, and even a necessary, step forward.

The second problem advanced by Güss et al. (2020) refers to the quality of police data itself. In the present commentary, we will try to add some general and fundamental aspects of police reports that were not mentioned by Güss et al. The following issues may be of relevance to illustrate the specific conditions of the genesis of police reports. The expectation of obtaining free access to high-quality police data for research purposes may be somewhat naïve. In applied research, one might typically expect a scientist–practitioner gap as in other applied disciplines, i.e., clinical psychology or industrial-organizational psychology (Anderson et al., 2010; Aguinis et al., 2017). This gap refers to the poor connection between scientific research generated by academia and the use of this research evidence by practitioners in the field: there is a “mindset clash” between police practitioners and academic researchers. Applied police research has to address differences between science and practice, whereas science needs to achieve the state of pragmatic science with both high methodological rigor and high practical relevance, while police practice needs to be evidence-based (Sherman, 1998; Anderson et al., 2010).

## Specialty of Police Reports

Among many complex factors with the police, the following issues may be of relevance to the researcher–practitioner gap concerning police science: Among many complex factors, the following issues may be of relevance to the researcher–police practitioner gap:

(a) The self-conception of police organizations differentiates from that of scientific organizations. Beside legal restrictions, for instance regarding data protection restricting data access, police organizations do not consider themselves as scientific institutions or “think tanks.” In the course of organizational socialization, police officers originally adopt a mindset as practitioners with less emphasis on scientific rigor or scientific requirements. Thus, the original purpose of police reports is not to provide information for researchers. Although investigative proceedings using police reports, such as the application of criminalistics methods, forensics, and the writing of sound legal reviews, are a highly demanding part of the job, police reports are primarily written with respect to the specific job demands of investigative proceedings rather than the demands of the social sciences.
(b) Güss et al. (2020) stated that police reports might be biased, while pointing out some highly relevant aspects of bias. Indeed, we would assume that bias itself determines the nature of police reports; they are not objective records of what happened within police operations. Rather, police reports show the underlying cognitive bias and memory susceptibility of those who generate them (Tversky and Kahneman, 1974; Loftus, 1979; Loftus and Pickrell, 1995; Lewinski et al., 2016; Loftus et al., 2021). Police reports are initially the outcome of behavioral observations, forensics, and interviews or interrogations conducted by the police officers themselves. Here, the competencies, work experience and even word choice of police officers performing investigative interviews and interrogations might affect the data collection, which forms the basis of the subsequent police reports (e.g., Loftus and Palmer, 1974; Loftus, 1975; Shaw and Porter, 2015; Thielgen et al., 2022). Secondly, the written product is predominantly a memory report with a time delay, at least to some extent. There are many possibilities for bias within interviewing and memorizing, and different factors can impact them (Cochran et al., 2016). In particular, confirmation bias may play the most important role (Nickerson, 1998). According to Nickerson (1998, p. 175–176), confirmation bias refers to the human “tendency to search for and interpret information in ways that are partial to existing hypotheses,” while ignoring opposing information to one's hypotheses or interpreting opposing information in terms of one's hypotheses. As Kassin et al. (2013, p. 45) noted, “[…] we use the term forensic confirmation bias to summarize the class of effects through which an individual's preexisting beliefs, expectations, motives, and situational context influence the collection, perception, and interpretation of evidence during the course of a criminal case.” This kind of information processing results in selective perception and interpretation of the environment (Lidén et al., 2018, 2019; Lidén, 2020), which is also a factor present in police officers starting the course of a criminal case with police reports. Usually, police officers are educated to search for both incriminating and exculpatory evidence. However, either the conviction of guilt or the presumption of innocence might be prevalent within police officers and hence control investigations, for instance in terms of biased information seeking. In addition, the police officers may actively themselves decide to write or not write a police report, depending on their evaluation of available information about putatively harmless incidents. Presumably, this self-evaluation tendency may also be generalized to other incidents.
(c) In recent police research, the role of police officers' mindset as a warrior or guardian has been repeatedly discussed (Conti, 2011; Rahr and Rice, 2015; Stoughton, 2016; McLean et al., 2019). This attitudinal dichotomy represents opposing cultural orientations of militarization and community policing within the police (Koslicki, 2020). Police officers who adopt a guardian mindset prefer service-oriented community policing based on principles of procedural justice. They commit to procedural justice procedures emphasizing cooperation, communication, and community within police operations. Their authority is perceived as based on consent with the public. In contrast, police officers adopting a warrior mindset view themselves as crime fighters, favoring an aggressive style of policing. In police–citizen encounters, they advocate a strict “us vs. them” mentality, relying on a need for authority to be asserted (Boivin et al., 2020). As police officers' mindsets seem to affect their behavior within police–citizen encounters, it may be assumed that the content of police reports may also be affected by the cultural orientations of police officers writing the reports (McLean et al., 2019; Carlson, 2020; Koslicki, 2020; Clifton et al., 2021). Officially, police leadership proclaim the police as a citizens' police (termed “Bürgerpolizei” in Germany), consisting of guardians which serve the public. Nevertheless, individual police officers might act as warriors aiming to engage in rigorous crime-fighting. Police reports may be adopted as a means to an end, e.g., to make sure that the suspect will punish for a crime of which the police officer is convinced.
(d) The role of police officers within police operations can be manifold. Presumably, police officers' role in investigative proceedings may fundamentally affect the police reports about an incident. Police officers can be initially directly involved in the police operation. For instance, they have to use a taser or gun, if attacked by an offender; perhaps they suffer an injury. Despite their active involvement, they can be eyewitnesses of relevant actions, for instance, if a colleague is attacked by an offender. Finally, they can be an interviewer of participants and witnesses. In the latter case, they usually record the information they evaluate as relevant during an operation and write down the police report at the end of service based on memory and notes. Police reports may vary depending on the police officers' role. In contrast to written police reports, one may also discuss the role of body-worn cameras filming police operations, CCTV material and video-taped interrogations.
(e) Furthermore, the nature of the incident and emotional involvement may impact the police reports (e.g., Honig and Lewinski, 2008; McClure et al., 2019). Specifically, rare and severe accidents or assaults (e.g., murder) with a high level of emotionality and stress levels may induce more distortion of information processing than more frequent and less severe incidents (e.g., material damage; Hulse and Memon, 2006; Zimmerman and Kelley, 2010). In contrast, one can assume that police officers may report the crime scene better than civilians based on their work knowledge and work routine (Christianson et al., 1998). Most prominent in this regard may be the weapon focus effect. As eyewitnesses, police officers are also prone to this memory bias. According to Fawcett et al. (2013, p. 2), the weapon focus effect “shall be defined as an object-related decrease in memory performance (e.g., feature or identification accuracy) for those elements of an event or visual scene coinciding with the presence of the weapon or unusual object” (e.g., Loftus et al., 1987). In a crime scene, police officers may tend to focus their attention on a present weapon and fail to encode information about the perpetrator's physical appearance and the surrounding context, subsequently failing to remember the scene as accurately as would have been the case had no weapon been visible. Consequently, it can be assumed that police reports about such incidents involving visible weapons may differ from those without any visible weapons, for example in terms of richness of detail.
(f) Despite some efforts at academization (e.g., police studies ending with a Bachelor degree in Germany), police studies in Germany mainly lack, or have less, methodological rigor compared to the scientific reasoning and scientific writing found in the social sciences. With respect to differences between the federal states, the value of scientific evidence and scientific methodology may presumably be underrated. Both scientific reasoning and scientific writing may not be sufficiently represented in the curricula of degree programs and do not play an important role within police studies. In the subsequent police practice, the training of police practitioners may direct them away from established evidence-based practices rooted in the scientific literature, especially that of the social sciences. Consequently, police reports are not scientific papers. In sum, a lack of evidence-based practice may exist in policing (Sherman, 1998; McDonnell et al., 2012; Lum and Koper, 2017, 2021).
(g) Although police officers might acknowledge the beneficial impact of the contribution of the social sciences to practical police work, scientific research as a knowledge process is perceived by police practitioners or police leaders to have little to no practical relevance to police work “on the streets.” Scientific processes (hypothesizing, reasoning, writing, and debating) may be viewed with skepticism or suspicion because they are often time-consuming, controversial, and exhausting to implement. However, police work is quite different and police reports depend on certain tasks. For instance, within criminal investigations of a murder, police officers might implicitly use a kind of scientific methodology when hypothesizing about the criminal act.
(h) The official language of police practitioners is more administrative than scientific with an emphasis on legal categories and terms. Due to time pressure and workload, “police language” may often operate with keywords and abbreviations. As a form of administrative communication, police reports serve as legwork for the prosecution and build up the foundation of legal proceedings. In this regard, one might expect that police officers would avoid self-incrimination, if there is a possibility that they have acted illegally. For instance, police use of force is illustrated from a police officer's perspective, neglecting the citizen's perspective in order to be legally correct.

## Discussion

In sum, police reports are a specific data source generated under specific conditions. Cognitive bias and memory malleability may mostly determine the quality of police reports, indicating that they are subjective measures. Additionally, threat and danger to life may have an impact on police officers' physical and mental functionality. Therefore, we suggest examining police reports as a research subject, questioning the factors that affect the content and quality of the reports. Police reports might be characterized by typologies of impact factors such as criminal acts or police officers' role and mindset.

Aside from cognitive bias and heuristics within police officers, there are several societal and cultural differences between Germany and the United States of America that may affect police reporting in general. For instance, in the United States of America the individual ownership of weapons is constitutionally warranted by the Bill of Rights. Consequently, the accessibility of weapons may be facilitated compared to Germany. It can be assumed that the availability of weapons among citizens may affect both the tactical approach of police officers within police operations and the subsequent police reports. According to the Washington Post's database, 1,022 people have been shot and killed by police in 2020 compared to 15 people in Germany (Tuason and Güss, 2019; Lorei, 2021; Washington Post, 2022). The number of fatal police shootings in the United States of America may indicate police brutality while black Americans are killed at a much higher rate than white Americans. This ethnicity bias may also be present in police reports pointing out the aforementioned issues. However, the racial bias in terms of discriminating against ethnic minorities does not seem to be limited to the United States of America (Vomfell and Stewart, 2021).

Addressing the problem of bias, police education and training may play an important role (Fridell, 2017). Within pedagogy the anti-bias curriculum of Derman-Sparkes (1989, p. 3) refers to as “an active/activist approach to challenging prejudice, stereotyping, bias, and the ‘isms.' In a society in which institutional structures create and maintain sexism, racism, and handicappism, it is not sufficient to be non-biased (and also highly unlikely), nor is it sufficient to be an observer. It is necessary for each individual to actively intervene, to challenge and counter the personal and institutional behaviors that perpetuate oppression.” Hohensee and Derman-Sparks (1992, p. 2) suggest specific learning goals of anti-bias curriculum in early childhood. Because of their universal validity, these goals may be applicable for police education: “(a) construction of a knowledgeable, confident self-identity, (b) comfortable, empathic interaction with people from diverse backgrounds, (c) critical thinking about bias, (d) ability to stand up for herself or himself, and for others, in the face of bias” (Derman-Sparks et al., 2020). Hence, self-esteem, empathy, critical thinking, and procedural justice are core prerequisites of anti-bias police work. The procedural justice approach of police training aims to shift police officers' attention away from crime control, surveillance, and punishment toward a democratic model of policing comprising respectful police-citizen-cooperation, public trust, and de-escalating communication (Tyler, 2003, 2006, 2011; Lum and Nagin, 2017; see also Nagin and Telep, 2020). Research found 4 principles of procedural justice that help to gain public trust and collaboration of citizens: First, police officers need to allow citizens to tell their story giving the chance to realize impact on police officers' behavior. Second, police officers need to make decisions fairly by making sure that citizens understand their explanations for decisions based on facts. Third, police officers need to behave respectful, polite, and honest. Finally, police officers need to concern the needs of citizens involved (Goodman-Delahunty, 2010; Tyler and Murphy, 2011; Mazerolle et al., 2013; Tyler et al., 2015).

According to social learning theory (Bandura, 1969, 1977, 1986, 1997) pedagogical professionals and leadership as role models obtain extraordinary importance within police. Furthermore, contact hypotheses of social psychology posits that interpersonal contact between in-group and outgroup members can effectively reduce intergroup conflict (e.g., between police and citizens) caused by bias, stereotypes, and discrimination (Allport, 1954; Pettigrew and Tropp, 2006). Thus, police education and training need to include the intergroup contact of police officers with minorities and crime victims, e.g., those suffering from police use of force.

Although police training emphasizing de-escalating communication, procedural justice, and anti-bias decision-making are highly desirable, research has provided little evidence on their behavioral effectiveness or on their unintended side effects so far (e.g., Lai et al., 2016; Forscher et al., 2019; McLean et al., 2020). Future research is needed to establish effective police training reducing the negative effects of bias and heuristics in police work and highlighting long-term effects on police officers' behavior (Birzer and Tannehill, 2001; Birzer, 2003).

Presumably, one of the most powerful tools of reducing bias is self-reflection (e.g., Lilienfeld and Landfield, 2008). According to Boud et al. (1985, p. 19) reflection refers to as “those intellectual and affective activities in which individuals engage to explore their experiences in order to lead to new understandings and appreciations” (Boud and Walker, 1990, 1998). Schön (1983, 1987) originated the most influential approach to reflection. His theory of reflective practice differs between reflection during the event in everyday practice termed as reflection-in-action, and reflection after the event termed reflection-on-action. The reflection-in-action approach seems to be applicable to dynamic events of police operations, see also Kolb (1984)'s reflective cycle, and the critical thinking approach proposed by Brookfield (1987).

## Author Contributions

SS developed the overview of the presented ideas and wrote the manuscript of the paper in English language. Both authors contributed equally to editing the first draft to its final version.

## Conflict of Interest

The authors declare that the research was conducted in the absence of any commercial or financial relationships that could be construed as a potential conflict of interest.

## Publisher's Note

All claims expressed in this article are solely those of the authors and do not necessarily represent those of their affiliated organizations, or those of the publisher, the editors and the reviewers. Any product that may be evaluated in this article, or claim that may be made by its manufacturer, is not guaranteed or endorsed by the publisher.
